# *Deep Consensus*, a deep learning-based approach for particle pruning in cryo-electron microscopy

**DOI:** 10.1107/S2052252518014392

**Published:** 2018-10-30

**Authors:** Ruben Sanchez-Garcia, Joan Segura, David Maluenda, Jose Maria Carazo, Carlos Oscar S. Sorzano

**Affiliations:** a Biocomputing Unit, Spanish National Center for Biotechnology, Calle Darwin 3, 28049 Madrid, Spain

**Keywords:** cryo-EM, deep learning, image processing, particle pruning, three-dimensional reconstruction

## Abstract

Single-particle cryo-electron microscopy (cryo-EM) has recently become a mainstream technique for the structural determination of macromolecules. Typical cryo-EM workflows collect hundreds of thousands of single-particle projections from thousands of micrographs using particle-picking algorithms. However, the number of false positives selected by these algorithms is large, so that a number of different ‘cleaning steps’ are necessary to decrease the false-positive ratio. Most commonly employed techniques for the pruning of false-positive particles are time-consuming and require user intervention. In order to overcome these limitations, a deep learning-based algorithm named *Deep Consensus* is presented in this work. *Deep Consensus* works by computing a smart consensus over the output of different particle-picking algorithms, resulting in a set of particles with a lower false-positive ratio than the initial set obtained by the pickers. *Deep Consensus* is based on a deep convolutional neural network that is trained on a semi-automatically generated data set. The performance of *Deep Consensus* has been assessed on two well known experimental data sets, virtually eliminating user intervention for pruning, and enhances the reproducibility and objectivity of the whole process while achieving precision and recall figures above 90%.

## Abstract 2

*Deep Consensus* performs particle pruning in cryo-EM image-processing workflows using a smart consensus.

## 1. Introduction   

The advent of direct electron detectors, together with the development of novel image-processing algorithms, have brought about a revolution in the field of single-particle analysis (SPA) in cryo-electron microscopy (cryo-EM; Nogales, 2016 ▸). As a result, SPA cryo-EM has been used to determine the structures of a wide range of protein complexes at near-atomic resolution (Bartesaghi *et al.*, 2015 ▸; Merk *et al.*, 2016 ▸; Banerjee *et al.*, 2016 ▸) and is now regarded as a very promising tool for computer-aided drug design (Rawson *et al.*, 2017 ▸).

In order to reconstruct the three-dimensional structure of a macromolecular complex employing SPA cryo-EM, tens of thousands of high-quality single-particle projections of the complex, generally termed particles, are required. These particles are picked from the micrographs, which generally suffer from a low signal-to-noise ratio, contamination and several other artifacts. These problems, along with the huge number of particles that need to be picked, make manual selection a tedious, time-consuming and potentially error-prone step, thus demanding substantial time and user intervention in most cases. On the contrary, automatic particle-picking algorithms (Adiga *et al.*, 2005 ▸; Sorzano *et al.*, 2009 ▸; Voss *et al.*, 2009 ▸; Abrishami *et al.*, 2013 ▸; Scheres, 2015 ▸; Wang *et al.*, 2016 ▸) are well suited for high-throughput workflows, being able to quickly collect thousands of particles with decent performance. Nevertheless, the set of false-positive particles that are selected by these automatic methods is non-negligible, typically ranging from 10% to more than 25% (Zhu *et al.*, 2004 ▸). As a consequence, it is common practice in the field to perform several pruning steps (Razi *et al.*, 2017 ▸; Aramayo *et al.*, 2018 ▸) in which a combination of algorithmic approaches such as particle sorting (Vargas *et al.*, 2013 ▸) or two-dimensional classification (de la Rosa-Trevín *et al.*, 2013 ▸; Scheres, 2012 ▸; Kimanius *et al.*, 2016 ▸; Yang *et al.*, 2012 ▸) are employed together with manual intervention in order to rule out false-positive particles.

One such approximation is *MAPPOS* (Norousi *et al.*, 2013 ▸). In order to use *MAPPOS*, a training set of true particles and false particles needs to be provided by the user. The particles are then described by a set of seven features such as phase symmetry or dark dot dispersion. Finally, a bagging classifier of different types of algorithms, including decision trees and *k*-nearest neighbors, is employed to predict which already picked particles are good and which are false positives. Although this methodology showed promising results, it has not been commonly adopted, probably because the task of manually picking several hundred of particles, although doable, is regarded as a time-consuming task and thus other approaches are used instead. Moreover, the features that are employed to describe particles can suffer from various shortcomings (Vargas *et al.*, 2013 ▸), limiting its applicability.

Recently, there has been a major breakthrough in machine learning with the development of deep learning (LeCun *et al.*, 2015 ▸; Krizhevsky *et al.*, 2012 ▸). Deep-learning approaches have shown outstanding performances in many different tasks such as image recognition, natural language processing and language translation, outperforming not only classical machine-learning algorithms but also humans in some tasks (He *et al.*, 2015 ▸). The main difference of deep learning with respect to classical machine-learning approaches is its ability to learn directly from raw data, which makes the labor-intensive design of handcrafted features unnecessary (LeCun *et al.*, 2015 ▸). Some of the most popular deep-learning models are convolutional neural networks (CNNs), which are the state-of-the-art in artificial vision (http://www.image-net.org/challenges/LSVRC). CNNs are multilayered feed-forward neural networks which are fed directly with input images. A typical CNN has two types of layers: convolutional and fully connected layers. Convolutional layers consist of convolution and pooling steps in tandem. The convolution kernels are automatically learned from training data and the pooling steps perform a down-sampling of the convolution outputs. Finally, the fully connected layers are classical neural network layers, the very last one of which produces an output that measures, in a classification problem, the probability of the input belonging to a given class.

Following this line of investigation, in this work we present *Deep Consensus* (*DC*), a deep learning-based approach for the problem of particle pruning. Our method employs a deep CNN designed to classify which of the already picked particles are true particles and which are false positives. In this development, we pursued three main objectives: to rule out false particles, while retaining most of the good particles, to perform this efficiently and, finally, to reduce manual supervision to very low levels. In order to train our CNN, a data set of true and false particles needs to be elaborated. Although this could be compiled manually, here we propose a semi-automatic approach for the generation of such a training set. This latter procedure is based on the fact that different particle-picking algorithms work on different principles, so that the particles that they select may be substantially different. This consideration would naturally suggest the exploration of meta classifiers; this is performed in this work while everything is simultaneously posed in a deep-learning framework. Instead of considering particles selected by just one single picker, we propose considering all particles selected by any of the picking algorithms as candidates and letting the CNN automatically decide which particles are good independently of the algorithm that picked them. *DC* was evaluated using two well known data sets, EMPIAR-10028 (ribosome; Wong *et al.*, 2014 ▸) and EMPIAR-10061 (β-galactosidase; Bartesaghi *et al.*, 2015 ▸), achieving precision and recall figures above 90%. The *DC* method is publicly available through the *Scipion* (de la Rosa-Trevín *et al.*, 2016 ▸) and *Xmipp* 3 (de la Rosa-Trevín *et al.*, 2013 ▸) cryo-EM frameworks.

## 2. Methods   

### 2.1. Algorithm   

**Figure 1:**
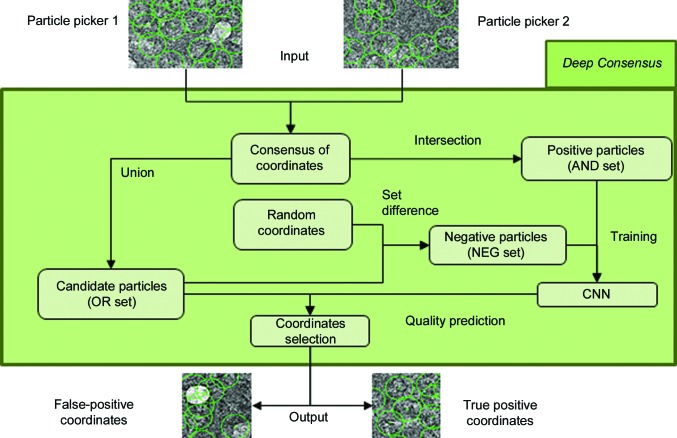
*Deep Consensus* workflow. *Deep Consensus* takes the coordinates proposed by different particle pickers as input, from which the intersection (AND set) and the union (OR set) of these coordinates are computed. Next, it picks random coordinates providing that they do not overlap with the OR set (NEG set). The NEG and AND sets are then used to train a convolutional neural network (CNN) that will finally classify the coordinates of the OR set (which is the largest set) as positive particle coordinates or negative particle coordinates.

The *DC* method takes the particles picked by several algorithms as input and produces a new set of high-quality particles as output. To this end, *DC* employs a CNN trained on a set of positive and negative particles collected from the outputs of several particle pickers (see Fig. 1 ▸). Specifically, the intersection of several sets of particles is defined as the set of particles with common coordinates in each set and is used as a positive training set, termed the AND set (see Section 2.3 for further details). Any particle contained in the AND set has been picked by all of the pickers and, as a consequence, the number of false positives included in this set is expected to be smaller than in any of the single-particle sets. However, the expected low false-positive rate of the AND set comes at the cost of reducing the number of selected particles. Similarly, the union of particles, termed the OR set, is defined by the union of particles that have been picked by any of the methods. This set will contain many false positives but also most of the true, and thus useful, particles. The negative particles (termed the NEG set) used for training are picked at random coordinates, ensuring that there is no particle of the OR set that is close to the randomly picked particle. Additionally, as randomly picked particles tend to overrepresent empty or uncentered particles, a set of negative particles comprised of ice, carbon and other undesired types of contamination can be manually included in the NEG set (see Section 2.3 for further details). Finally, once the CNN has been trained using the AND and NEG sets, the particles of the OR set are evaluated and a score ranging from 0 to 1 is assigned to each particle. In the absence of any *a priori* information, especially if the user wants to run *DC* in a fully automated manner, particles with scores of greater than or equal to 0.5 will be classified as true positives, whereas particles with scores below 0.5 will be classified as false positives and thus removed from the final set. At this point, it is worth noting that better thresholds can be manually chosen after visual inspection of the final ranking.

It is important to note that even after the careful automatic construction of the training sets described above, it is a fact that nothing is perfect and that there will be errors in the positive and negative training sets (the AND and NEG sets, respectively). Fortunately, it is well known that deep-learning approaches tend to be very robust to label noise (Rolnick *et al.*, 2017 ▸; Zhang *et al.*, 2017 ▸; Jindal *et al.*, 2016 ▸). As a consequence, our CNN is indeed able to learn useful information from the AND and NEG sets, and the resulting pruned set is more accurate than any of the original input sets proposed by the particle pickers (see Appendix *C* [App appc]). Moreover, as the number of putative particles (the OR set) is also larger than the individual sets obtained from each picker, a larger number of positive particles can also be recovered while still maintaining low false-positive particle levels, which results in substantial gains in speed and reproducibility with respect to workflows that do not use *DC* (see Section 3.2).

### 2.2. Deep convolutional neural network   

The core component of *Deep Consensus* is its CNN. CNNs are very popular deep-learning models that have shown outstanding performance in many artificial vision problems. For a brief introduction to neural networks and CNNs, see Appendix *A* [App appa].

The architecture of our deep CNN, which we derived after a thoughtful cross-validation process, is summarized in Table 1 ▸. Our model, implemented using *Keras* 2.1.5 (https://github.com/keras-team/keras) and *TensorFlow* 1.4 (Abadi *et al.*, 2016 ▸), consists of four blocks of two convolutional layers followed by a batch normalization (Ren *et al.*, 2017 ▸) and a pooling (down-sampling) layer. The output of the last convolutional block is fed into a single fully connected layer. Finally, a SoftMax layer is employed to obtain the probability of an input being either a good particle or a bad particle. Regularization is performed using a dropout with *p* = 0.5 after the fully connected layer, and L2 weight regularization was applied to each layer with strength 1 × 10^−5^. All layers were initialized using the default mechanisms included in *Keras*.

Network training is performed using the *Adam* optimizer (Kingma & Ba, 2014 ▸) and cross entropy as a loss function with default parameters and an initial learning rate of 1 × 10^−4^ until convergence is detected. A validation set is drawn from the training set, collecting a random sample of 10% of the set. When the validation accuracy does not improve for 300 steps (batches), the learning rate is divided by ten. Data augmentation is performed using random flips and rotations over the training set at a 1:1 ratio.

### 2.3. Training-set collection: consensus of coordinates and random picking   

Given several sets of coordinates picked by different particle pickers, *Deep Consensus* (*DC*) firstly computes the intersection and the union of these sets of coordinates (the AND and OR sets, respectively). The OR set is composed of all of the coordinates picked by any picker. The AND set is obtained considering the Euclidean distances between each coordinate of the first set and all of the coordinates of the second set; if the distance between a particular particle is smaller than the 10% of the particle size, the average co­ordinate is added to the AND set. When more than two sets of coordinates are used, the previous strategy is repeated considering the coordinates of the AND set and the coordinates of the third set, then the fourth set, and so on.

The set of negative particles used for training (the NEG set) is collected from randomly picked coordinates. As some of these coordinates may correspond to actual particles, we remove those coordinates that are close (closer that 50% of the particle size) to any coordinate included in the OR set. We filter out coordinates using the OR set instead of the AND set because there are many more true-positive particles in the OR set than in the AND set, and we prefer to ensure that most of the particles included in the NEG set are negative particles. Random picking strategies tend to obtain mostly empty particles or noncentered particles. However, particles containing ice or picked in the carbon regions are underrepresented. For this reason, we allow the user to provide a complementary set of negative particles, such as that available at http://campins.cnb.csic.es/deep_cons, that will be merged with the NEG set. In the case that the user does not provide such a set, as in the examples presented in this work, we alternatively employed a set of negative particles that was semi-automatically collected by applying *Xmipp* particle sorting to the AND set and considering particles with *Z*-scores in the top 1%, which typically correspond to the worst picked particles.

### 2.4. Evaluation: testing-set elaboration   

In order to evaluate the performance of our method, we employed precision-recall and receiver operating characteristic (ROC) curves and computed the areas under both curves for each data set. Similarly, we estimated the accuracy, precision, recall and Matthews correlation coefficient (MCC) of *DC* predictions using the value that maximizes the MCC as a threshold. Such evaluations require a testing data set of particles for each evaluated case. We elaborated these by employing the particles deposited in EMPIAR as positive particles and randomly picked particles as negative particles, as performed in training-set generation. With the aim of improving the quality of our testing set, for each complex we manually curated both the positive and the negative particles of the initial testing set to a subset of 2000 positive and 2000 negative particles in which we also included ice, contamination *etc.* similarly as was performed in training-set generation.

### 2.5. Evaluation: resolution estimation   

The goodness of the pruning performed by *DC* was also assessed by estimating the resolution values reached by the selected particles. To this end, we employed the *RELION* *auto-refine* algorithm (Scheres, 2012 ▸; Kimanius *et al.*, 2016 ▸) using the set of particles that we collected internally in *DC* (AND/OR sets) as input as well as the *DC*-selected set (*DC*-retained set) and the filtered-out set of particles (*DC*-pruned set). The initial volumes required for *auto-refine* were downloaded from EMDB (Tagari *et al.*, 2002 ▸) entries EMD-2984 and EMD-2984 filtered at 60 Å. In order to obtain the *DC*-retained and *DC*-pruned sets, a threshold of 0.5 was employed. Consequently, the *DC*-retained set is composed of all of the particles originally found in the OR set with a *DC* score greater than or equal to 0.5 and the *DC*-pruned set contains the particles included in the OR set with *DC* scores smaller than 0.5.

### 2.6. Evaluation: two-dimensional classification and class averages of different particle sets   

The class averages displayed in Section 3.1.3 were obtained using the *RELION* two-dimensional classification algorithm (Scheres, 2012 ▸; Kimanius *et al.*, 2016 ▸) on the particles of the OR, AND and *DC*-retained sets (a threshold of 0.5 was set). We only show the four classes with the most particles. All 32 classes obtained in the execution of the *RELION* two-dimensional classification are included in Appendix *B* [App appb].

### 2.7. Comparison with other pruning approaches   

*DC* was compared with other pruning methodologies in terms of achieved resolution and execution time. Resolution estimation was performed as explained in Section 2.5. Two-dimensional classification was performed using *RELION*-2.0. Other two-dimensional classification algorithms such as *cl*2*d* in *Xmipp* (Sorzano *et al.*, 2010 ▸) or *ISAC* in *SPARX* (Yang *et al.*, 2012 ▸) were also initially considered, but since they were much slower than *RELION*-2.0 on GPUs and their use was only for comparison purposes and was not at the core of our newly proposed method, they were not finally included in this work. Both *RELION* two-dimensional classification and *DC* were executed using one Nvidia 1070 GTX graphics card, while *RELION* *auto-refine* was executed using two Nvidia 1070 GTX graphics cards. *DC*-retained particles were selected with a 0.5 threshold.

## 3. Results and discussion   

### 3.1. Performance evaluation   

The performance of our approach has been assessed on two publicly available data sets: EMPIAR-10028 (ribosome; Wong *et al.*, 2014 ▸) and EMPIAR-10061 (β-galactosidase; Bartesaghi *et al.*, 2015 ▸). In both cases we compiled the *DC* input sets using two well established algorithms: the *Xmipp* autopicker (Abrishami *et al.*, 2013 ▸) and the *EMAN*2/*SPARX* Gaussian picker (Tang *et al.*, 2007 ▸; Hohn *et al.*, 2007 ▸). We carried out three different quality-assessment tests on the obtained results: a statistical analysis of manually curated testing sets of particles, a visual inspection of two-dimensional class averages and an evaluation of the final resolution obtained from the different sets of particles.

#### 3.1.1. Statistical analysis   

**Figure 2:**
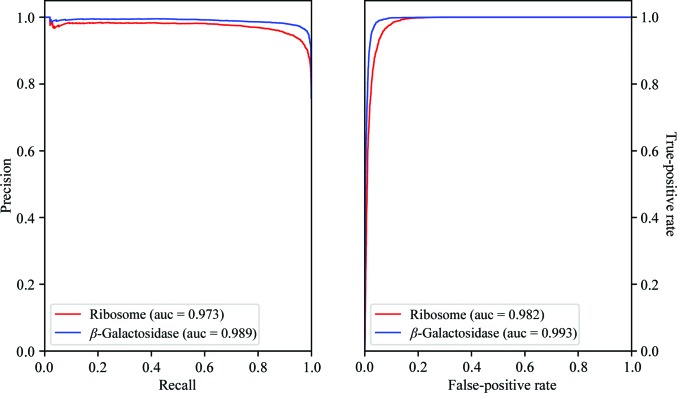
*Deep Consensus* precision-recall and ROC curves computed from testing sets. Red, the EMPIAR 10028 data set (ribosome); blue, the EMPIAR 10061 data set (β-galactosidase). The area under the curve (auc) is given in parentheses.

We evaluated the performance of *DC* by measuring several statistical scores on a manually curated testing set derived from particles deposited in the EMPIAR database (Iudin *et al.*, 2016 ▸; see Section 2.4 for details of the testing-set collection) and we compared it with the individual performance of each of the particle pickers that were used for input to *DC*. Table 2 ▸ displays how *DC* obtained almost perfect classification metrics for the two evaluated data sets, with precision and recall values above 0.9. Similarly, the curves displayed in Fig. 2 ▸ are close to a perfect classification, indicating that *DC* was able to distinguish positive particles from negative particles outstandingly well. Finally, as can be appreciated from Table 2 ▸, *DC* achieved better results than those obtained by the individual particle pickers used as input, which proves that *DC* has succeeded in going beyond merely combining the two sets of input particles.

#### 3.1.2. Resolution analysis   

At this point, we wanted to better understand the new scenario that *DC* brings to the SAP field with a very simple and direct experiment: what are the final resolutions of the different cryo-EM maps obtained from each of the particle sets? For simplicity, we always use the same reconstruction method (Scheres, 2012 ▸; Kimanius *et al.*, 2016 ▸) with all parameters set to the same values for the different runs (see Section 2.5). In this way, the single difference between the runs will be the different data sets of particles that are being fed into the algorithm. Table 3 ▸ compares the different resolution values computed over the different particle sets.

From inspection of this table, it is very clear that the resolution obtained using the OR set of particles (those picked by at least one of the pickers) is comparable or worse than the resolution obtained using the AND set (the consensus data set among the pickers), even if this latter set contains an order of magnitude fewer particles than the former set. However, many good particles are missing in the small AND set, which is shown clearly by the fact that if we were to use the results of only ‘the best picker’ (we refer to the picker that performed the best in these two data sets, which was the *Xmipp* picker) the resolution would still be better than that obtained with the small AND particle set.

However, the power of *DC* becomes evident when viewing the results presented in the two leftmost columns. Indeed, when the particles retained by *DC* are employed, the resolution of the maps always increases over that achieved when using any other data set, including that formed by the particles from the best-performing picking algorithm. At the same time, it is very clear that those particles rejected by *DC* (which are between 20% and greater than 30% of the OR data set) were creating very important inconsistencies in the whole reconstruction process, simply because their resulting map has a resolution that is an order of magnitude worse than that obtained with any other data set.

Finally, if we also consider the number of particles included in the AND, OR and *DC*-retained sets, it seems clear that our method has been able to go beyond the AND set by rescuing from the OR set many particles that are not included in the AND set but are still positive particles and thus good candidates to achieve a better resolution map. This also proves that our neural network, which has been trained on a reduced and slightly mislabeled training set, is able to learn and generalize to other particles that were not included in the training set.

#### 3.1.3. Two-dimensional classification and class averages of different particle sets   

**Figure 3:**
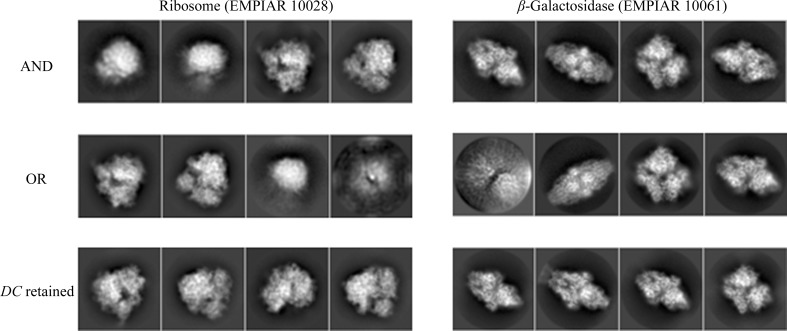
Averages of the major classes obtained from the particles in the AND, OR and *DC*-retained data sets, all computed using the *RELION* two-dimensional classification algorithm.

In order to obtain another assessment of the value of *DC*, we performed a two-dimensional classification using the same data sets as presented in the previous section and compared the classes visually. As before, we used the same processing parameters in all runs, only changing the data sets. Fig. 3 ▸ shows the four most populated two-dimensional class averages obtained from the particles in the AND, OR and *DC*-retained data sets, so that a visual (qualitative) assessment can be performed (the averages of all classes have been included in Appendix *B* [App appb]). Within the limits of this assessment (and considering that the four major classes from each data set do not necessarily have to present the complex in the same orientation, especially for data sets with a good angular coverage, as is the case in our examples), at the level of high-resolution content the classes obtained from the *DC*-retained particles are the best, followed by those from the AND data set, while those from the OR data set are very poor. These results support the notion that *DC* has been able to prune many of the bad particles contained in the OR set but yet recovered many more particles than those included in the AND set.

### 3.2. Comparison with other pruning approaches   

In SPA cryo-EM there is currently a trend towards selecting as many particles as possible at the beginning of the image-processing workflow (Wang *et al.*, 2016 ▸) despite the inclusion of many false positives. These false-positive particles are expected to be ruled out in the subsequent steps of the image-processing workflow, especially in the very time-consuming steps of two-dimensional and three-dimensional classification, which are certainly demanding of computational resources but also rely on strong human intervention, a factor that introduces subjective decisions into the analysis workflow. Consequently, and this is one of the motivations of this work, processing workflows can be substantially accelerated and automated by applying precise pruning methods such as *DC* at the very beginning of the image-processing workflow in such a way that the initial set of particles will be smaller but better.

In order to grasp how useful our approach may be, we have compared *DC* with other techniques that are commonly employed as pruning steps, specifically *Z*-score particle sorting (Vargas *et al.*, 2013 ▸) and two-dimensional classification (Scheres, 2012 ▸; Kimanius *et al.*, 2016 ▸). Comparisons have focused on the execution time and on the quality of the selected particles (see Section 2.7 for details).

Owing to their similarities, we would have liked to compare *DC* with *MAPPOS* (Norousi *et al.*, 2013 ▸); however, as neither the program nor the particles that were used to evaluate *MAPPOS* are available, we were not able to do so. Nevertheless, it is worth noting that our approach exhibits a very important advantage over *MAPPOS*: the semi-automatic training-set generation. Moreover, the precision-recall values reported in the *MAPPOS* publication (around 0.9 and 0.7, respectively) are noticeably worse than those that we have measured in this work and, although not directly comparable, they are in line with the idea that deep-learning algorithms tend to outperform conventional machine-learning approaches.

When *DC* was compared with the *Z*-score particle-sorting approach (Vargas *et al.*, 2013 ▸), using default thresholds for both methods, *DC* was able to prune many more particles. Moreover, the resolution obtained by *DC* was substantially better than that obtained when using only *Z*-score particle sorting.

A more detailed comparison was performed between the relative performance of *DC* and a ‘typical’ step of particle pruning by two-dimensional classification. Naturally, pruning by two-dimensional classification requires user intervention, which in the case of the results presented in this manuscript was achieved by a member of the laboratory experienced in dealing with many experimental data sets. Still, there is an intrinsic ‘human variability and subjectivity’ factor in this procedure that is unavoidable (and that *DC* aims to abolish). In this way, *DC* was able to prune slightly more particles than two-dimensional classification, while preserving essentially the same resolution values (see Table 4 ▸). Similarly, in order to study the composition of the ruled-out sets of particles, we measured the resolution that was achieved by these sets using the same evaluation procedure for both *DC* and two-dimensional classification (see Table 2 ▸). Thus, we measured a resolution of ∼12 Å for the EMPIAR-10061 data set (12.7 and 11.4 Å, respectively), whereas a resolution of ∼32 Å was measured for the EMPIAR-10028 data set (33.5 and 31.6 Å, respectively); the resolution was better for EMPIAR-10061, probably because the set of discarded particles was larger, but was still similar for both *DC* and two-dimensional classification. Accordingly, from the ‘final resolution’ point of view, *DC* and pruning by two-dimensional classification achieved similar results, suggesting that they could be employed interchangeably. However, under our benchmarking conditions (see Section 2.7) the running time of *DC* was approximately five times faster than a typical two-dimensional classification step while not introducing any human subjectivity, leading to automation; these are two very valuable parameters in order to increase data processivity and reproducibility in cryo-EM.

## 4. Conclusions   

In this work, we describe *Deep Consensus*, a deep-learning approach for the pruning of cryo-EM particles. Our method employs a convolutional neural network (CNN) trained on a set of true and false particles. Contrary to most deep-learning setups, for which data sets need to be carefully compiled by human experts, we employed a semi-automatically collected training set. This training set is obtained by computing the consensus outputs of multiple particle-picking algorithms. Thus, the positive particles used for training are obtained by taking the intersection of the output coordinates (allowing some error margins), whereas the negative particles are picked at random coordinates that are distant from any of the particles picked by any method (union). Finally, the particles contained in the union are classified as positive or negative by the trained CNN and those classified as negative are removed from the initial set.

We have shown that the *Deep Consensus* approach is considerably robust to mislabeling, and thus our method is able to be trained using sets that contain a significant fraction of false positives and false negatives. Indeed, as shown in Section 3.1, we have proven that *Deep Consensus* was able to learn from the semi-automatically generated sets and was able to identify a large number of positive particles that were not included in the initial training set, while removing many of the negative particles selected by the picking algorithms.

Finally, we have compared *Deep Consensus* with other pruning strategies and showed that it works better, or at least as well, as commonly applied methods. Moreover, *Deep Consensus* provides an important advantage, since it accelerates the image-processing workflow while avoiding user subjectivity, so that it can be fully standardized and automated. As a result, *Deep Consensus* seems to be a very promising approach for application at the very beginning of cryo-EM workflows, just after the step of particle picking.

Consequently, we consider that we have achieved our aim of helping the user to accelerate and automate one of the most critical steps in cryo-EM image processing, particle pruning, thus increasing objectivity and reproducibility in the initial steps of processing and at the same time facilitating the widespread usage of cryo-EM image-processing workflows by users with many diverse backgrounds.

*Deep Consensus* is publicly available from *Xmipp* (http://xmipp.cnb.csic.es) and *Scipion* (http://scipion.cnb.csic.es).

## Figures and Tables

**Figure 4:**
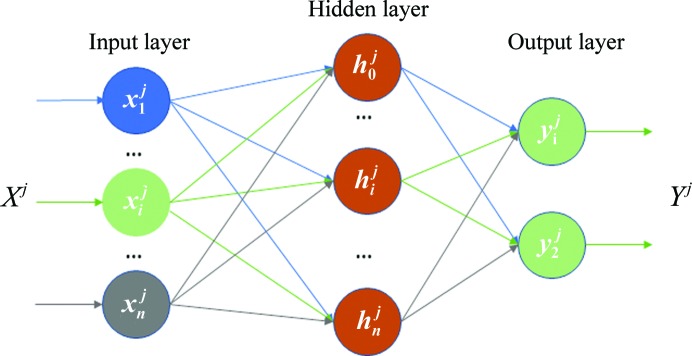
Artificial neural network for binary classification. *X^j^* = (*x*_1_^*j*^, *x*_*i*_^*j*^, …, *x*_*n*_^*j*^) is one example to be classified by the network. *h*_*i*_^*j*^ is the output of a hidden neuron, computed as shown in (1). *Y^j^* = (*y*_1_^*j*^, *y*_2_^*j*^) is the output of the network for the example *X^j^* computed using (2).

**Figure 5:**
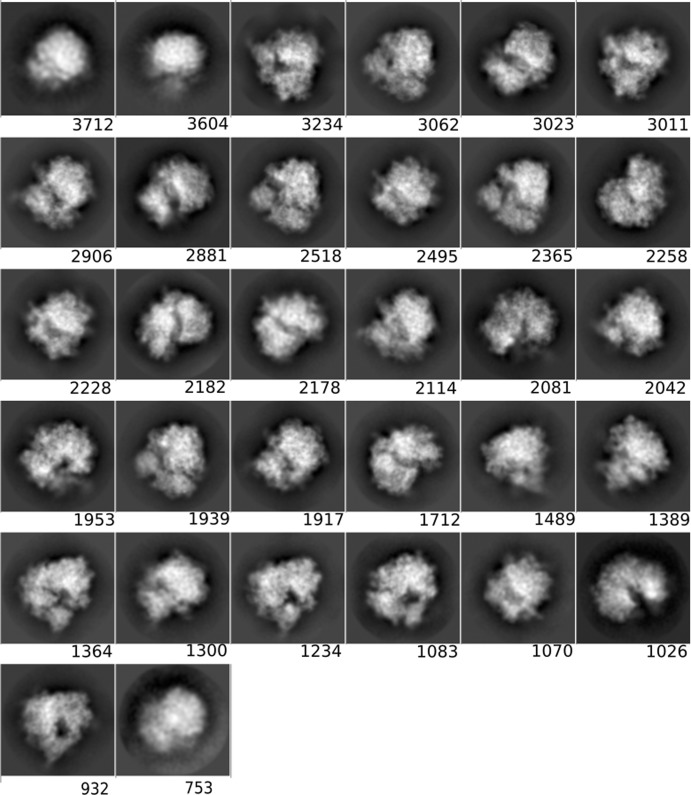
Class averages for the AND set (picked by all particle pickers).

**Figure 6:**
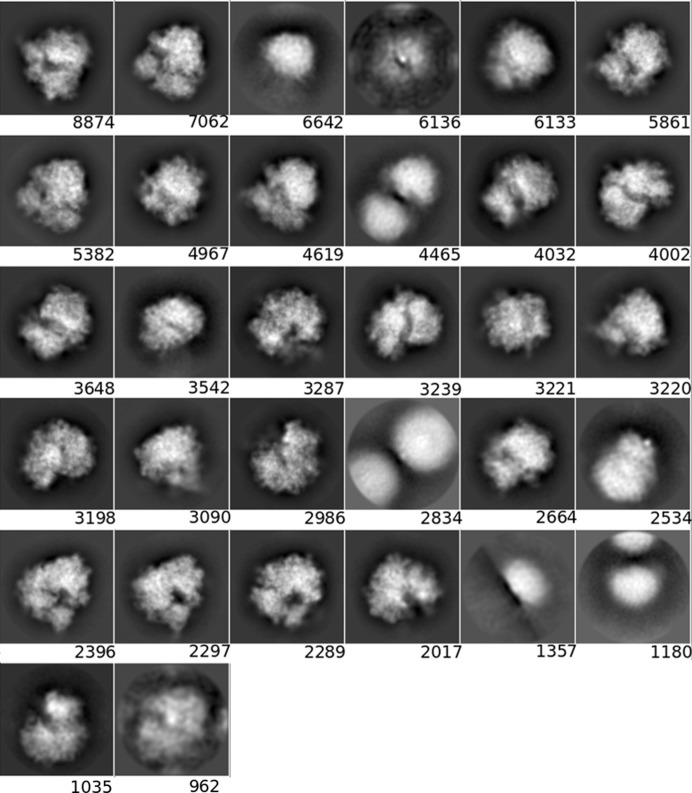
Class averages for the OR set (picked by any particle picker).

**Figure 7:**
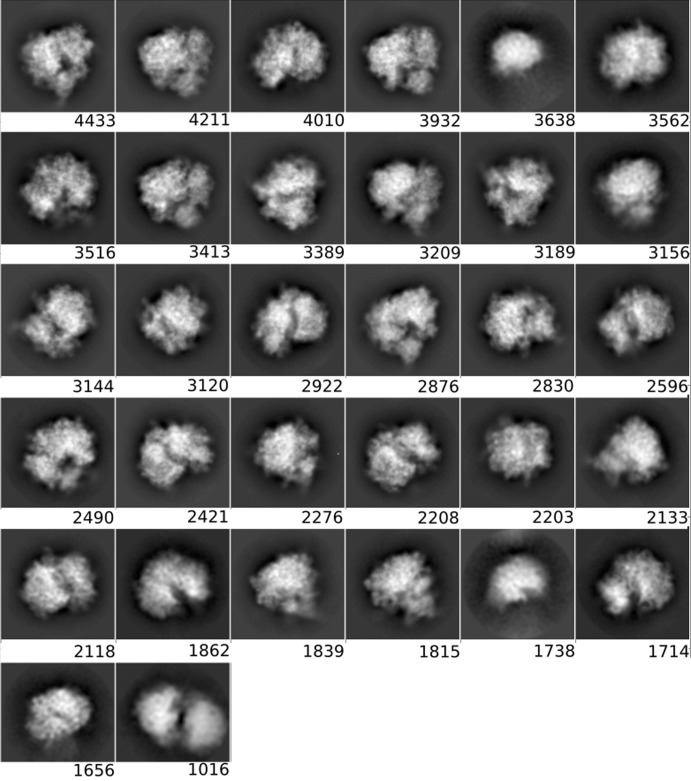
Class averages for the *DC*-retained set (selected by *Deep Consensus*).

**Figure 8:**
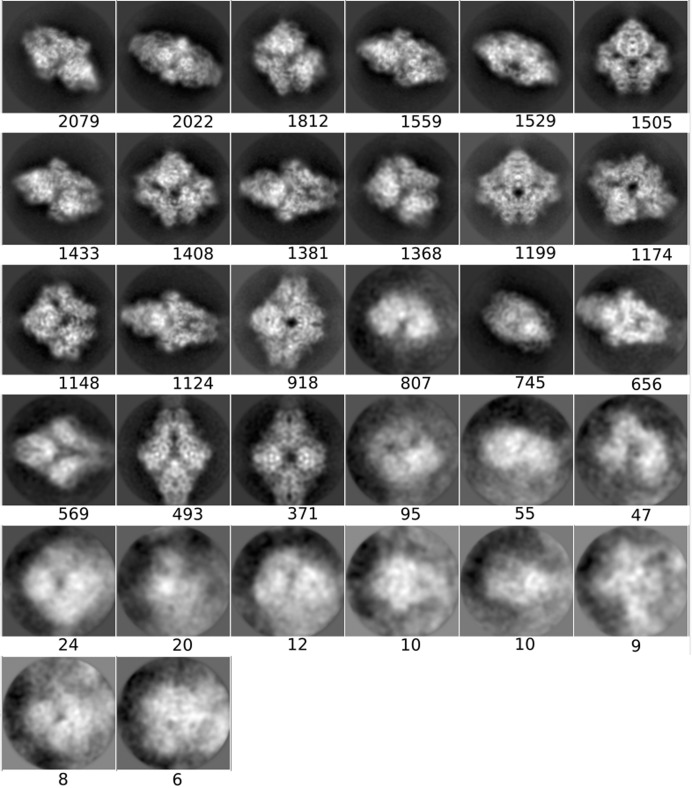
Class averages for the AND set (picked by all particle pickers).

**Figure 9:**
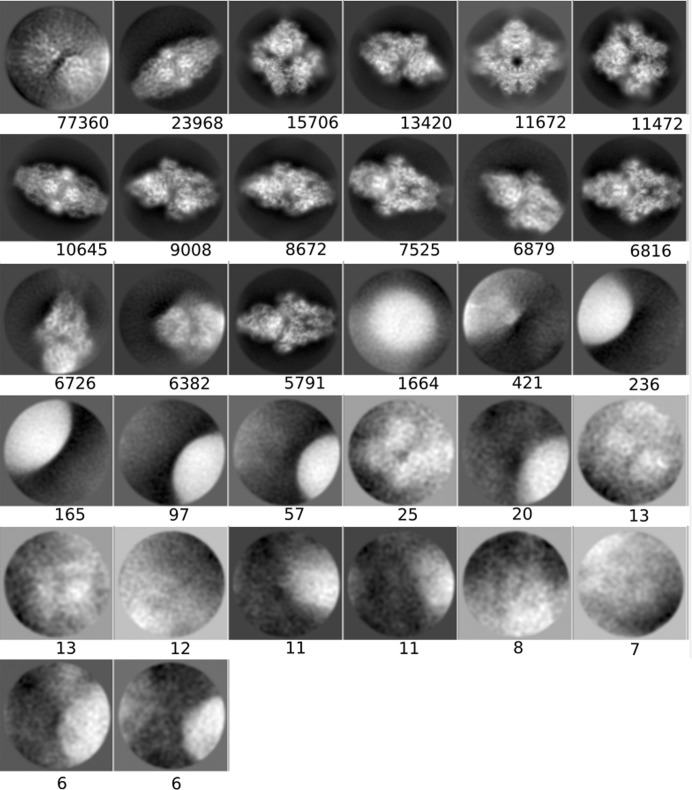
Class averages for the OR set (picked by any particle picker).

**Figure 10:**
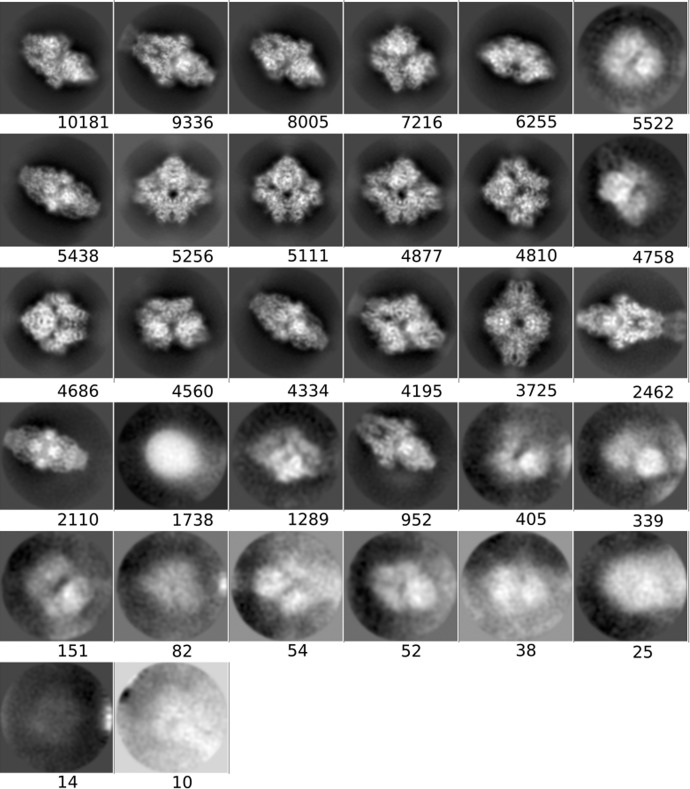
Class averages for the *DC*-retained set (selected by *Deep Consensus*).

**Table 1:** The deep convolutional neural network architecture employed in *Deep Consensus*

Layer No.	Layer type	Kernel size/step size	Shape
1	Input	—/—	128 × 128 × 1
2	Conv2D + relu	15/1	128 × 128 × 8
3	Conv2D + batch normalization + relu	15/1	128 × 128 × 8
4	MaxPooling2D	7/2	64 × 64 × 8
5	Conv2D + relu	7/1	64 × 64 × 8
6	Conv2D + batch normalization + relu	7/1	64 × 64 × 16
7	MaxPooling2D	5/2	32 × 32 × 16
8	Conv2D + relu	3/1	32 × 32 × 32
9	Conv2D + batch normalization + relu	3/1	32 × 32 × 32
10	MaxPooling2D	3/2	16 × 16 × 32
11	Conv2D + relu	3/1	16 × 16 × 64
12	Conv2D + batch normalization + relu	3/1	16 × 16 × 64
13	AveragePooling2D	4/2	8 × 8 × 64
14	FullyConnected + relu + dropout (*p* = 0.5)	512/—	512
15	SoftMax	512/—	2

**Table 2:** Statistical measurements of *Deep Consensus* and the performance of the input particle pickers MCC, Matthews correlation coefficient; ACC, accuracy; ROC-auc, area under the ROC curve; PR-auc, area under the precision-recall curve; NA, not available; DC, *Deep Consensus*; Xmipp, *Xmipp* autopicker; Gaussian, *EMAN*2/*SPARX* Gaussian picker. MCC, precision and recall were computed at the threshold that maximizes the MCC for DC and at the suggested threshold for Xmipp and Gaussian.

Algorithm	EMPIAR data set	MCC	ACC	Precision	Recall	ROC-auc	PR-auc
DC	10061	0.889	0.944	0.927	0.965	0.982	0.973
DC	10028	0.942	0.971	0.958	0.984	0.993	0.989
Xmipp	10061	0.782	0.893	0.898	0.849	NA	NA
Xmipp	10028	0.818	0.908	0.872	0.937	NA	NA
Gaussian	10061	0.697	0.845	0.778	0.904	NA	NA
Gaussian	10028	0.726	0.871	0.798	0.860	NA	NA

**Table 3:** Resolution achieved in both data sets when refining different sets of particles OR, particles selected by any picker; AND, particles selected by both pickers; BPP, particles selected by the picker that obtained the best results; *DC*-pruned, particles ruled out by *Deep Consensus*; *DC*-retained, particles selected as good by *Deep Consensus*; *R*, resolution; *N*, number of particles.

	OR		AND		BPP		*DC*-retained		*DC*-pruned	
EMPIAR data set	*R* (Å)	*N*	*R* (Å)	*N*	*R* (Å)	*N*	*R* (Å)	*N*	*R* (Å)	*N*
10061	3.76	231251	3.32	25600	2.92	117047	2.83	125586	12.74	105665
10028	3.83	119171	3.87	67043	3.70	97561	3.65	88622	33.50	30549

**Table 4:** Comparison of different pruning approaches *DC*-retained, particles selected as good by *Deep Consensus*; *Z*-score-retained, particles that were selected as good using *Xmipp* particle sorting; R-2D-retained, particles that were selected as good by an expert after using *RELION* two-dimensional classification; *R*, resolution; PR, percentage of retained particles compared with the total number of particles (231 251 and 119 171, respectively); *T*, running time of the pruning algorithm; TT, total running time for the pruning and *RELION* *auto-refine* steps.

	*DC*-retained				*Z*-score-retained				R-2D-retained			
EMPIAR data set	*R* (Å)	PR (%)	*T* (h)	TT (h)	*R* (Å)	PR (%)	*T* (h)	TT (h)	*R* (Å)	PR (%)	*T* (h)	TT (h)
10061	2.83	54.3	3.9	20.3	3.72	95.2	2.0	25.1	2.80	56.9	23.1	40.2
10028	3.65	74.4	2.7	12.0	3.77	94.9	1.4	14.6	3.65	85.6	14.8	27.6

**Table 5:** *Deep Consensus* performance on testing sets when trained using synthetic AND sets with different levels of mislabeling noise R, ribosome data set (EMPIAR-10028); G, β-galactosidase data set (EMPIAR-10061); MCC, Matthews correlation coefficient; ACC, accuracy; ROC-auc, area under the ROC curve. MCC, precision and recall were computed at the threshold that maximizes the MCC.

	MCC		Precision		Recall		ACC		ROC-auc	
Corruption level	R	G	R	G	R	G	R	G	R	G
0%	0.934	0.884	0.965	0.937	0.952	0.927	0.982	0.961	0.992	0.969
25%	0.906	0.861	0.946	0.928	0.948	0.914	0.965	0.950	0.987	0.967
30%	0.875	0.851	0.923	0.918	0.942	0.914	0.949	0.944	0.978	0.964
40%	0.722	0.672	0.845	0.834	0.872	0.786	0.870	0.849	0.926	0.878
45%	0.435	0.264	0.710	0.652	0.736	0.527	0.720	0.630	0.776	0.663
50%	0.087	0.077	0.563	0.574	0.598	0.646	0.536	0.531	0.523	0.491

**Table 6:** *Deep Consensus* precision and recall on testing sets when trained using synthetic AND sets of different sizes with different levels of mislabelling noise R, ribosome data set (EMPIAR-10028); G, β-galactosidase data set (EMPIAR-10061); #Partic, number of true particles included in the data set; corrupt, corruption level. Each cell displays the precision and recall measured in each condition.

#Partic	3000		2000		1000		500	
Corrupt	R	G	R	G	R	G	R	G
30%	0.923/0.942	0.918/0.914	0.920/0.923	0.902/0.917	0.866/0.926	0.876/0.952	0.800/0.888	0.816/0.900
40%	0.839/0.902	0.840/0.897	0.774/0.819	0.835/0.890	0.738/0.820	0.762/0.595	0.693/0.818	0.726/0.586
45%	0.705/0.762	0.695/0.817	0.662/0.698	0.610/0.701	0.602/0.777	0.581/0.731	0.625/0.709	0.578/0.604

## Appendix A An introduction to convolutional neural networks

Neural networks (NNs) are bioinspired machine-learning models that, after several years of modest use, have been rebirthed under the name deep learning. NN models are composed of several layers of neurons (also known as hidden units) that are connected one after another. Typically, NN architectures are constituted of an input layer, an output layer and several hidden layers in between (see Fig. 4 ▸). Each neuron of the input layer represents an input variable of a data example (for example the intensity of one pixel), whereas the neurons of the output layer produce the predictions associated with the data example. Each of the neurons of a hidden layer computes a weighted average of its inputs (*x*_1_, *x*_2_, …, *x*_*n*_) and applies a nonlinear function to it, typically relu (1). The inputs of a neuron are the outputs of other neurons, which come from the previous layer or directly from the input data if the neuron belongs to the first hidden layer. The output of the neural network is also a weighted average of the last hidden layer units (2), but in this case it is converted to probabilities using a SoftMax function (3).

The predictive power of an NN comes from its ability to learn from a labeled set of examples (a training set). During the NN training process, the weights associated with each neuron are calculated to minimize a cost function that measures the differences in the training-element labels and their predicted probabilities. For classification problems, such as that considered in this work, the cost function accounts for misclassification of the training examples [the difference between the neural network prediction *y*(*X^j^*) and the training label *l^j^* for an example *j*] and thus the model will tend to classify training examples correctly (4):

However, classifying training examples correctly does not guarantee that the model will perform well on data that have not being used for training. Indeed, this lack of generalization to new data when performance in the training set is good is known as overfitting and is a common problem that, until recent developments, had limited the applicability of NNs.

One of these developments is the convolutional neural network (CNN) architecture, which is currently the preferred choice when facing artificial vision problems such as image classification. The main difference of CNN convolutional layers (CNLs) with respect to regular NN layers, which are generally called fully connected layers (FCLs), is the connectivity pattern of the hidden neurons. Thus, whereas FCL neurons are connected to all neurons of the previous layer independently of the dimensions of the input, CNL neurons are locally connected to windows of neurons in the previous layers. As each neuron slides over all possible windows, these layers perform a convolution with self-learned kernels over the outputs of the previous layers, and this is the reason for the name of this architecture. This difference makes CNNs particularly well suited to image-related problems because the local connectivity of neurons in CNLs produces layer output images, and thus they retain the structure of the input through the different layers, as the input and the output of a CNL are both images. Additionally, the total number of parameters of a CNN is much smaller than the number of parameters of a fully connected network and thus it is less prone to overfitting, leading to better performance. For a complete review, see LeCun *et al.* (2015 ▸).

## Appendix B Class averages obtained from the different data sets using *RELION* two-dimensional classification

In this section we show the 32 class averages that were obtained after *RELION* two-dimensional classification executed with default parameters. Classes are sorted from major to minor. The number of particles that belong to each class is shown below each class image.

### Data set 1: EMPIAR 10028

Figs. 5 ▸, 6 ▸ and 7 ▸ show the class averages for the AND set (picked by all particle pickers), the class averages for the OR set (picked by any particle picker) and the class averages for the *DC*-retained set (picked by any particle picker), respectively, for this data set.

### Data set 2: EMPIAR 10064

Figs. 8 ▸, 9 ▸ and 10 ▸ show the class averages for the AND set (picked by all particle pickers), the class averages for the OR set (picked by any particle picker) and the class averages for the *DC*-retained set (picked by any particle picker), respectively, for this data set.

## Appendix C Tolerance of *Deep Consensus* to mislabeling

In order to understand how *DC* behaves when the training set is affected by mislabeling noise, we carried out a set of experiments in which the CNN that *DC* employs was trained using a synthetic training set (instead of the AND set) derived from a ground true set of true particles and false particles that had been manually curated. In each of the experiments, we introduced different levels of label corruption, understanding the corruption level as the fraction of particles that have been incorrectly labeled. The training set contained a total number of 3000 putative true particles and 3000 putative false particles, with all putative particles being correct in the case of 0% corruption and half of them being incorrect in the case of 50% corruption.

As can be appreciated from Table 5 ▸, *DC* shows a remarkable tolerance to mislabeling, achieving a similar performance at a corruption level of 25% to that obtained when no mislabeling is present. Similarly, it also seems clear that the statistics obtained for a corruption level of 30% are comparable to the statistics obtained for the single-particle pickers that we employed for the compilation of the AND set (see the main text and Table 2 ▸), and thus a 30% corruption level could be thought of as the failure threshold for the AND set in order to improve the results of the individual particle pickers. Fortunately, obtaining such accuracy levels for the AND set is theoretically easy because of the different natures of the different particle pickers. Indeed, the intersection of two independent partitions of a data set that display 50% accuracy each is expected to show 75% accuracy at the cost of halving the number of included particles. Of course, this theoretical calculation only holds for ideal systems, but it still helps to understand how the intersection allows better training sets to be obtained provided that the size of the intersection is large enough.

In our experiments, we have found that the intersection of the output of the two particle pickers can be relatively small compared with the total number of particles picked by each (data not shown). For this reason, we have also studied the impact of the number of particles on the performance of *DC* at different corruption levels. Table 6 ▸ shows the precision and recall estimated for each scenario. Two interesting conclusions can be drawn. Firstly, at a corruption level of 30%, *DC* is able to function well using a small training set of size 2000 (1000 positives and 1000 negatives). Secondly, the impact of mis­labeling noise can be severely reduced depending on the training-set size. This second conclusion, which is in line with that stated in Rolnick *et al.* (2017 ▸), together with the theoretical result mentioned above, provides the key to understanding the reliability of *DC*: the accuracy of the individual particle pickers is less relevant (to a certain level) than the size of the intersection. Consequently, when the intersection of the particle pickers represents an important fraction of the total number of picked particles, the results obtained by *DC* are expected to be good.
